# Dual-Modality Photoacoustic and Ultrasound Imaging System for Noninvasive Sentinel Lymph Node Detection in Patients with Breast Cancer

**DOI:** 10.1038/srep15748

**Published:** 2015-10-29

**Authors:** Alejandro Garcia-Uribe, Todd N. Erpelding, Arie Krumholz, Haixin Ke, Konstantin Maslov, Catherine Appleton, Julie A. Margenthaler, Lihong V. Wang

**Affiliations:** 1 Optical Imaging Laboratory, Department of Biomedical Engineering, Washington University in St. Louis, St. Louis, Missouri, USA; 2 Philips Research North America, Briarcliff Manor, NY, USA; 3 Department of Radiology, Washington University School of Medicine, St. Louis, MO, USA; 4 Department of Surgery, Washington University School of Medicine, St. Louis, MO, USA

## Abstract

The detection of regional lymph node metastases is important in cancer staging as it guides the prognosis of the patient and the strategy for treatment. Sentinel lymph node biopsy (SLNB) is an accurate, less invasive alternative to axillary lymph node dissection. The sentinel lymph node hypothesis states that the pathological status of the axilla can be accurately predicted by determining the status of the first lymph nodes that drain from the primary tumor. Physicians use radio-labeled sulfur colloid and/or methylene blue dye to identify the SLN, which is most likely to contain metastatic cancer cells. However, the surgical procedure causes morbidity and associated expenses. To overcome these limitations, we developed a dual-modality photoacoustic and ultrasonic imaging system to noninvasively detect SLNs based on the accumulation of methylene blue dye. Ultimately, we aim to guide percutaneous needle biopsies and provide a minimally invasive method for axillary staging of breast cancer.

Lymph nodes are small oval-shaped organs that are part of the body’s lymphatic system. They are found widely throughout the body and are connected to one another by lymph vessels. The sentinel lymph node (SLN) is defined as the first node in the lymphatic system that drains a tumor site. The sentinel lymph node concept is based on the finding that methylene blue dye (or other dyes and radioactive isotopes) is taken up into the lymphatic system similar to how tumor cells travel from the tumor. Thus, the first lymph node or cluster of lymph nodes where the dye is taken up represent the sentinel lymph nodes. Histopathologic and molecular assessment of the dissected sentinel lymph node has enhanced the detection of clinically occult nodal metastases^1^. A sentinel lymph node biopsy (SLNB) involves less extensive surgery and the removal of fewer lymph nodes than standard axillary lymph node dissection (ALND)^2^. A negative SLNB result suggests that cancer has not spread to nearby lymph nodes or other organs. A positive SLNB result indicates that cancer is present in the sentinel lymph node and may be present in other nearby lymph nodes and, other organs. The detection of regional lymph node metastases is both a major prognostic factor and a decision criterion for treatment in patients with breast cancer^3^,^4^,^5^. The SLNB sensitivity is more than 90%, with a specificity of 100%^6^,^7^. Besides the presence of metastasis or micrometastasis detected in the sentinel lymph node (SLN) after excision and histological examination, the total number of involved regional lymph nodes is important in staging the disease, with the number predicting overall survival with an inverse relationship^8^,^9^,^10^.

Ultrasonography (US) examination of the axilla has become common practice in the presurgical assessment in patients with a new diagnosis of invasive breast cancer at most academic institutions, including the Alvin J. Siteman Cancer Center (Washington University). US permits the visualization of lymph node size, shape, contour and changes in cortical morphology and texture that appear to be associated with the presence of axillary metastases^11^,^12^,^13^. However, ultrasonographic signs of metastatic disease sometimes overlap with those of benign reactive changes, limiting the ability of US to accurately stage the axilla. Further, US cannot differentiate SLNs from downstream lymph nodes. Often, the axillary US identifies normal-appearing lymph nodes. Needle biopsy is not performed in these instances because it is unknown if the normal-appearing lymph nodes represent the true SLN. In some patients, the axillary US identifies an abnormal-appearing lymph node. Image-guided fine needle aspiration biopsy (FNAB) is performed in these instances but only a positive result is informative. Negative needle biopsy does not rule out the presence of malignancy due to the possibility of sampling error and the inability of US alone to identify the SLN.

Typically, the conventional SLNB procedure consists of injecting radioactive tracers and/or methylene blue dye to mark the lymphatic system and guide the surgeon to the sentinel node^14^,^15^. The radioactive tracer is injected a few hours prior to the surgery, while methylene blue, which spreads relatively quickly through lymph vessels, is injected in the operating room. A few minutes following methylene blue injection, a surgical incision is made in the area indicated by a hand-held Geiger counter. The surgeon interrogates the axilla and identifies nodes that have been stained blue or nodes that are detected as radioactive with the Geiger counter. These nodes are then removed for histological examination to determine the presence of tumor metastases.

Here, we propose the use of dual-modality photoacoustic tomography (PAT)^16^,^17^,^18^,^19^ and US for accurate identification of SLNs. In PAT, an optical pulse is employed to irradiate an area of the body. The optical energy is absorbed rapidly by the tissue and resulting in the production of ultrasonic waves via the photoacoustic effect^20^. The image is reconstructed from the measured photoacoustic signals by an ultrasonic transducer placed outside the body. The PAT image visualizes the product of the spatially variant optical absorption coefficient (in units of m^–1^) and the local optical fluence (in units of J/m^2^).

Due to the scattering and absorbing nature of biological tissue, optical fluence decays rapidly with the increase in depth. As a result, the amount of light reaching a point far from the light source may become insufficient to detect deep SLNs. PAT combines the advantages of pure optical and ultrasonic imaging methods. First, PAT benefits from the tissue’s low acoustic scattering. Ultrasonic scattering in biological tissues is approximately three orders of magnitude less than optical scattering, which allows PAT to achieve high acoustic resolution at depths far beyond the optical diffusion limit^20^,^21^.

Consequently, PAT has higher spatial resolution in deep tissues than pure optical imaging, which relies on strongly scattered photons for the spatial resolution. Second, scattered light presents less difficulty to PAT because any absorbed light is converted to sound. Third, PAT is inherently compatible with US, thereby enabling dual-modality imaging with complementary contrasts. PAT has been shown to penetrate as deeply as 7 cm in tissue, which is sufficient for many clinical applications, such as breast imaging^22^. The US function of PAT-US enables the anatomical identification of lymph nodes, while the PAT function determines if the lymph node is a SLN by imaging accumulated blue dye.

Unlike US, PAT is highly sensitive to the strong optical absorption of the blue dye. An accurate identification of SLN by PAT-US can allow sampling of the SLN using fine needle aspiration biopsy (FNAB) for a minimally invasive approach to axillary staging^23^.

We developed a multi-modality PAT-US imaging based on a modified clinical US scanner (iU22, Philips Healthcare). A system diagram is shown in Fig. 1. The laser emitted pulses with a pulse duration of 6.5 ns at a repetition rate of 10 Hz. We employed methylene blue dye, which is routinely used in clinical practice, as our contrast agent. An optical wavelength near the peak absorption wavelength of methylene blue dye (667 nm) was chosen. All per-channel data from the US transducer were transferred to a custom-built data acquisition (DAQ) computer, which performed image reconstruction and displayed PAT, US, and co-registered images live at 5 frames per second.

## Results

**Figure 2:**
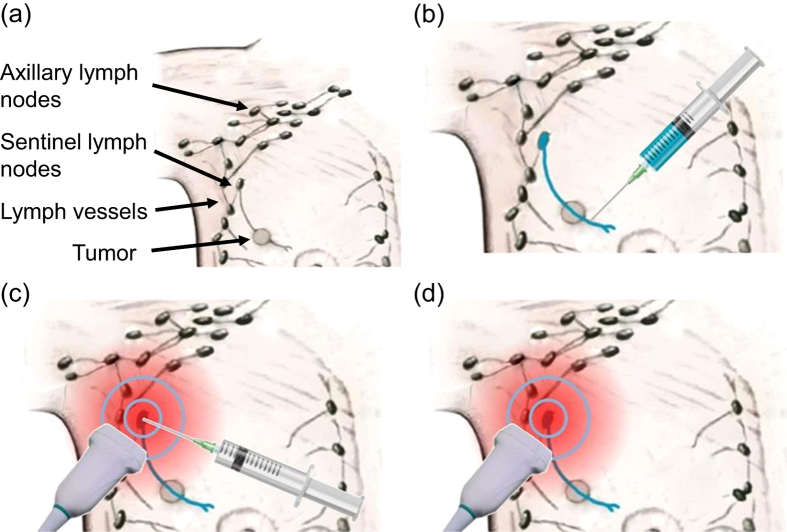
Overview of PAT-US imaging approach for minimally invasive SLN staging. (**a**) Anatomy showing the relevant structures. (**b**) Periareolar injection of methylene blue dye, which is collected by lymphatic vessels and drained to the sentinel lymph node. (**c**) PAT-US imaging pinpoints the SLN. (**d**) PAT-US imaging guides a FNAB of the SLN with high contrast.

The proposed staging method is illustrated in Fig. 2. First, study participants received a single subcutaneous injection of methylene blue dye near the areola in the same breast quadrant as the primary tumor. Since methylene blue is commonly used clinically for SLN biopsy, this new PAT method builds on the proven track record and physician’s experience with the dye for SLN detection. Next, real-time axillary PAT-US imaging is performed. Once US detects lymph nodes based on their characteristic appearance (hypoechoic outer cortex and hyperechoic central hilum), PAT distinguishes sentinel nodes from downstream nodes based on strong photoacoustic contrast from methylene blue. Once the suspected SLN was identified by the radiologist, a small tissue marker (commercially available titanium clip, routinely used in breast biopsy) was deployed under PAT-US guidance into the suspected SLN. Finally, PAT-US imaging was performed to confirm proper clip placement in the suspected SLN. Ultimately, PAT-US will be used to guide percutaneous FNAB to enable a minimally invasive approach for lymph node staging.

**Figure 3:**
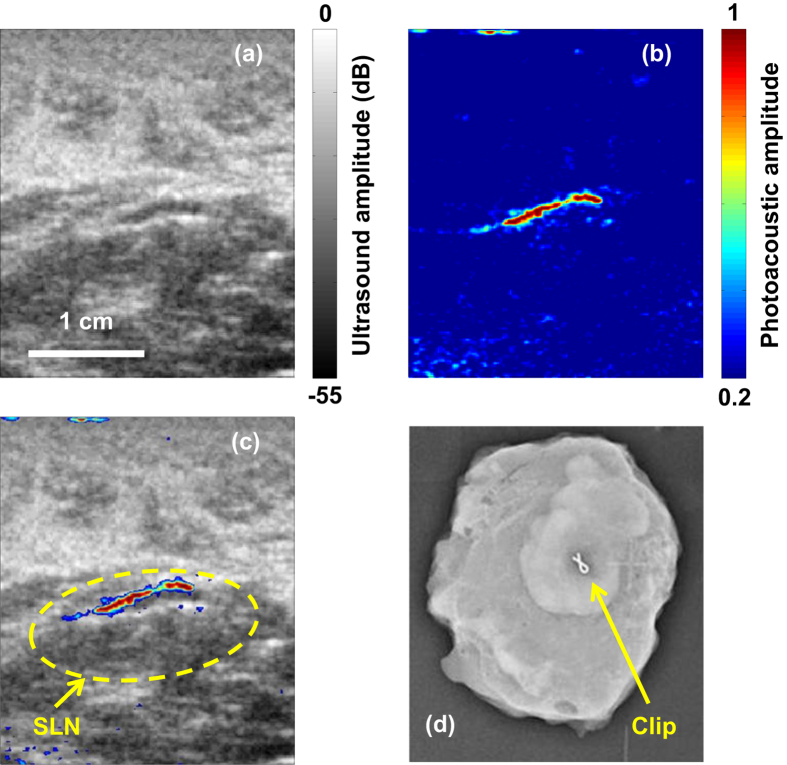
(**a**) *In vivo* US image of a lymph node. (**b**) *In vivo* PAT image of methylene blue dye. (**c**) Co-registered PAT-US image of the SLN. (**d**) Radiograph of *ex vivo* SLN showing the presence of the tissue marking clip.

Figure 3a,b show an example of corresponding US and PAT images, respectively, revealing a SLN (Movie S1). The co-registered PAT and US image (Fig. 3c) shows the capability of the technology to locate lymph nodes via anatomical features in US, and to verify the lymph node as a sentinel node using PAT. The SLN was marked *in vivo* by deploying a titanium clip under PAT-US guidance. Once the PAT-US procedures were completed, participants underwent conventional SLN biopsy immediately. Both methylene blue dye and radiocolloid were detected according to standard methods. The surgically removed SLNs were imaged with conventional specimen radiography to locate the titanium clips, and pathologically examined further to determine whether the tissue marker clip was present in the specimen. For this patient, the SLN identified during the standard surgical procedure was confirmed to be the same SLN identified by PAT-US (Fig. 3d).

**Figure 4:**
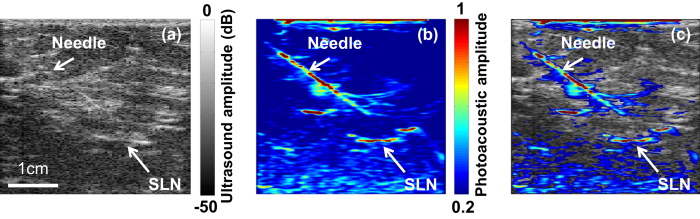
*In vivo* images of the SLN and the needle acquired using PAT-US. (**a**) *In vivo* US image showing the lymph node and needle. (**b**) *In vivo* PAT image of the SLN and needle. (**c**) Coregistered PAT-US image of the SLN and needle.

PAT-US can provide guidance for needle-based medical interventions^24^,^25^. For SLNB, PAT-US can serve as a real-time modality to first locate the SLN and then guide a fine or core needle for lymph node sampling. Figure 4a,b show the corresponding US and PAT images, respectively, revealing the SLN and the needle used to insert the titanium marking clip. The contrast from the needle in the PAT image is much higher than that in the corresponding US image because the ultrasound array receives more emitted photoacoustic energy than it does reflected ultrasonic energy. The co-registered PAT and US image (Fig. 4c, Movie S2) shows again the capability of the technology to locate lymph nodes via anatomical features in US and to verify the lymph node as sentinel using PAT. In addition, these results illustrate the feasibility of PAT-US to guide needles to the SLN in breast cancer patients.

Sixteen women were entered into this pilot clinical study, and two additional cases are excluded here due to detection system malfunction. All patients had pathologically proven breast cancer and were scheduled to have an axillary lymph node dissection. Twelve patients had clinically negative nodes in the axilla and four patients had clinically positive lymph nodes. A single tissue marker clip was placed in thirteen cases. The presence of a marker clip in the SLN specimen was confirmed in six cases.

## Discussion

In 2014, approximately 235,000 women developed breast cancer in the United States^26^. A large number of these patients may be eligible for a staging axillary lymph node dissection. The financial costs of cancer are high for both the person with cancer and for society as a whole. A simpler and less costly technique has the potential for considerable savings of costs in addition to morbidity. The goal of this clinical study was to develop a technique to minimize the morbidity and costs associated with staging axillary lymph nodes in patients with breast cancer. The results from this study are an important step toward clinical translation of photoacoustic imaging for minimally invasive SLN detection.

Several factors can affect the ability to successfully identify SLNs, such as insufficient network of lymphatics at the site of injection to accumulate an adequate amount of methylene blue. Also, single-wavelength PAT, while highly sensitive to methylene blue contrast, is not specific to methylene blue. In some cases, the high sensitivity to methylene blue was sufficient to overcome the lack of specificity. In these cases, the high signal-to-noise ratio and contrast from the blue stained node was such that the SLN was easily detected. Nevertheless, confounding photoacoustic signals can be problematic. PAT is sensitive to hemoglobin absorption in blood vessels as well. As lymph nodes are vascularized structures, the feeding and draining blood vessels can be sources of PAT contrast, which must be distinguished from methylene blue.

**Figure 5:**
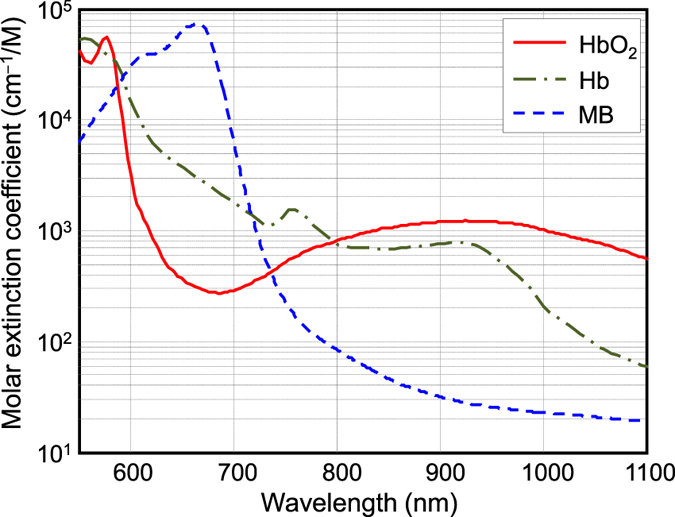
Molar extinction coefficient of oxy-hemoglobin (HbO2) and deoxy-hemoglobin (Hb) as well as methylene blue (MB) dye.

SLN identification can be improved by adopting multi-wavelength PAT to spectrally resolve methylene blue from blood in nearby vessels. By tuning the excitation light to multiple wavelengths, the photoacoustic signal varies according to the absorption spectrum of the target. Methylene blue absorption is strongest at 665 nm, while its absorption is very weak at wavelengths longer than 750 nm^27^ (Fig. 5). Spectral differences between methylene blue and hemoglobin can be exploited by using two wavelengths; one wavelength near the peak optical absorption of methylene blue (i.e., 665 nm) and a second wavelength where methylene blue absorption is minimal (i.e., >740 nm).

Dual-wavelength PAT was implemented on the prototype PAT-US system by interleaving laser pulses from the Nd:YAG laser with pulses from the dye laser. The fundamental Nd:YAG wavelength (1064 nm) was a good choice for spectral separation of methylene blue dye and blood without the need of an additional laser. The PAT-US imaging system was able to display the PAT image at 10 frames per second, while the excitation wavelength alternated between 1064 nm and 650 nm on consecutive laser shots. In addition, the methylene blue signal was highlighted for the physicians by employing a form of image subtraction (i.e., fractional change), which was displayed side-by-side with the live PAT image. Both the live PAT image and the fractional change image were displayed in real time at 10 frames per second.

**Figure 6:**
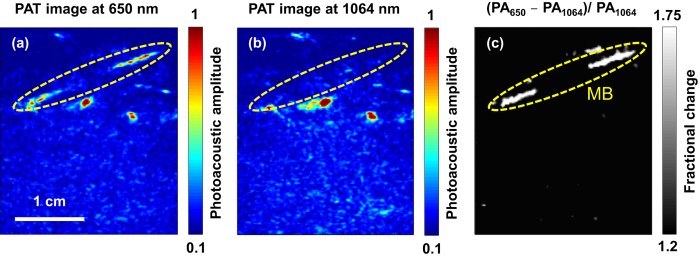
*In vivo*, dual-wavelength PAT. (**a**) PAT image acquired at 650 nm showing both methylene blue dye in lymphatic vessels and hemoglobin in blood vessels. (**b**) PAT image acquired at 1064 nm showing primarily hemoglobin in blood vessels while suppressing the signals from methylene blue dye. (**c**) Image displaying the fractional change (650 nm signal relative to 1064 nm signal) convincingly highlights the locations of methylene blue dye. PA_650_ and PA_1064_: photoacoustic amplitudes measured at 650 nm and 1064 nm, respectively.

Figure 6 shows an example of *in vivo* imaging of methylene blue and the ability of the system to distinguish methylene blue from surrounding blood vessels. Dual-wavelength PAT started five minutes after methylene blue injection and breast massage. The PAT image acquired at 650 nm (Fig. 6a) shows signals from both methylene blue (i.e., lymphatic vessels) and blood vessels, while the PAT image acquired at 1064 nm (Fig. 6b) primarily shows signals from blood vessels only. Figure 6c displays the image representing the fractional change (650 nm image relative to 1064 nm image), which convincingly highlights methylene blue (Movie S3). While the clinical utility of the dual-wavelength imaging requires further investigation, these initial results are promising and suggest that confounding signals from surrounding blood vessels can be removed effectively. More robust SLN detection would likely be enabled by employing dual-wavelength imaging given its much improved imaging specificity for methylene blue.

We successfully developed a dual-modality imaging system that integrates PAT and US. Our results show that co-registered PAT and US imaging can detect SLNs and lymphatic vessels using methylene blue dye. This technique is highly translatable since PAT can be added to conventional US systems, and methylene blue is already used clinically in the United States during SLNB. This approach offers significant benefits over the current standard of care; specifically, it would avoid the morbidity of the surgical SLNB technique and eliminate the need for radioactive isotopes in lymph node staging. Combined PAT and US-guided FNAB of SLNs potentially can eliminate the need for invasive axillary staging procedures.

## Methods

### Photoacoustic imaging system

A prototype system capable of performing PAT-US imaging was developed around a modified clinical US scanner (iU22, Philips Healthcare). The light source consisted of a wavelength tunable dye laser (PrecisionScan-P, Sirah, Kaarst, Germany) pumped by a Q-switched Nd:YAG laser (QuantaRay PRO-350-10, Spectra-Physics, Santa Clara, CA). The laser pulse was coupled to a fused-end, bifurcated fiber bundle that flanked both sides of a commercially available ultrasonic transducer array probe (L12-5 and L8-4, Philips). The fiber bundles were enclosed in a custom-made shell surrounding the transducer array and electronics to provide an integrated unit for easy, ergonomic, handling. The laser repetition rate was 10 Hz with a pulse duration of 6.5 ns. The optical fluence on the skin was less than 10 mJ/cm^2^, which was within the ANSI safety limit by a factor of 2^28^. The per-channel data was transferred to a custom-built data acquisition (DAQ) system for image reconstruction and display. The DAQ system was synchronized with laser firings by an FPGA-based electronic board that also performs PAT image reconstruction based upon a delay-and-sum beamforming algorithm. The system was capable to display PAT, US, and co-registered images live at 5 frames per second.

### Participant population

Participants were recruited from the patient population attending the Joanne Knight Breast Health Center of Washington University’s School of Medicine. Eligibility included having an age of 18 or older, the capability of informed consent, and newly diagnosed clinical stage I, II, or III breast cancer, and a planned surgery of their proven breast carcinoma. Additionally, the participant must have negative axillae, confirmed both by clinical exam and by directed ultrasound evaluation performed by an attending radiologist. Exclusions included contraindications for breast surgery, history of allergic reactions attributed to methylene blue dye or other agents used in this study, and uncontrolled intercurrent illness including, but not limited to, ongoing or active infection of the breast and/or axilla, symptomatic congestive heart failure, unstable angina pectoris, cardiac arrhythmia, or psychiatric illness/social situations that would limit compliance with study requirements.

### Patient preparation

Prior to imaging, the target area of the participant’s skin was prepared. Typically, any excessive amount of hair in the area to be imaged was removed. Methylene blue dye (5 mL, 2 mg/mL) was injected subcutaneously with a 25 gauge needle into the same breast quadrant as the primary tumor. The breast was massaged for 5 minutes to accelerate lymphatic flow. Methylene blue dye drains into the sentinel lymph node within minutes of injection.

All methods and experimental procedures were carried out in accordance with the approved guidelines of The Institutional Review Board of The Washington University School of Medicine. All experimental protocols were approved by The Institutional Review Board of The Washington University School of Medicine. All participants signed informed consents before were included in the study.

## Additional Information

**How to cite this article**: Garcia-Uribe, A. *et al*. Dual-Modality Photoacoustic and Ultrasound Imaging System for Noninvasive Sentinel Lymph Node Detection in Patients with Breast Cancer. *Sci. Rep.* **5**, 15748; doi: 10.1038/srep15748 (2015).

## Supplementary Material

Supplementary Information

Supplementary Movie S1

Supplementary Movie S2

Supplementary Movie S3

## Figures and Tables

**Figure 1:**
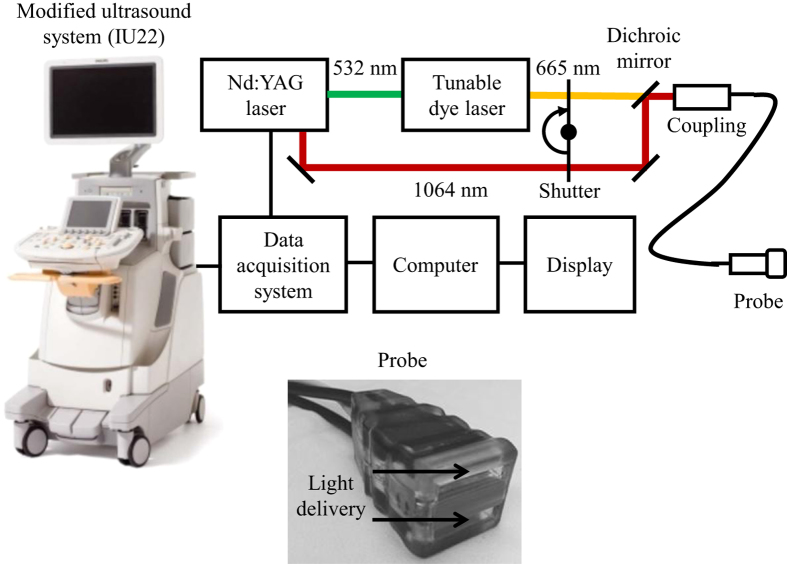
Schematic of the dual-modality photoacoustic and ultrasonographic imaging system and photograph of the probe. The probe features a flat surface for light delivery, and the fiber bundles are integrated into the ultrasonic transducer housing for ergonomic handling.
