# Supplementary material for: Dual-Modality Photoacoustic and Ultrasound Imaging System for Noninvasive Sentinel Lymph Node Detection in Patients with Breast Cancer
Source: Sci Rep. 2015 Oct 29;5:15748. doi: 10.1038/srep15748 (PMC4625171; doi:10.1038/srep15748)
Supplement: Supplementary Information [file srep15748-s1.doc]

Dual-Modality Photoacoustic and Ultrasound Imaging System for Noninvasive Sentinel Lymph Node Detection in Patients with Breast Cancer

Alejandro Garcia-Uribe^1^, Todd N. Erpelding^2^, Arie Krumholz^1^, Haixin Ke^1^,

Konstantin Maslov^1^, Catherine Appleton^3^, Julie A. Margenthaler^4^, Lihong V. Wang^1, *^

*^1^Optical Imaging Laboratory, Department of Biomedical Engineering, Washington University in St. Louis, St. Louis, Missouri, USA*

*^2^Philips Research North America, Briarcliff* *Manor, NY, USA*

*^3^Department of Radiology, Washington University School of Medicine, St. Louis, MO, USA*

*^4^Department of Surgery, Washington University School of Medicine, St. Louis, MO, USA*

*^*^* Corresponding author. E-mail: lhwang@wustl.edu.

**Supplementary Video 1 Caption**

An *in vivo*, simultaneously acquired PAT and US movie of a lymph node in a woman with breast cancer. The left and middle panels correspond to US and PAT, respectively. The right panel shows co-registered PAT-US images of the SLN. These images were displayed in real-time during the clinical procedure.

**Supplementary Video 2 Caption**

Co-registered PAT (in color) and US (in gray) images of SLN and a needle in a human patient acquired *in vivo*. The first part of the movie shows the localization of the SLN, the second part shows the deployment of a small tissue marker (a titanium clip) under PAT-US guidance into the suspected SLN.

**Supplementary Video 3 Caption**

*In vivo*, dual-wavelength PAT in human patient following methylene blue injection. The left panel shows the PAT images acquired at 650 nm, which represent signals from both methylene blue (i.e., lymphatic vessels) and blood vessels. The middle panel—the PAT images acquired at 1064 nm—shows primarily signals from blood vessels. The right panel displays the fractional change (650 nm signals relative to 1064 nm signals), which highlights the locations of methylene blue.
